# Cigarette and Hookah Smoking in Adolescent Students using World Health Organization Questionnaire Global Youth Tobacco Survey (GYTS): A Pilot Study in Varamin City, Iran in 2016

**DOI:** 10.31557/APJCP.2020.21.10.3033

**Published:** 2020-10

**Authors:** Mohammad Reza Masjedi, Elaheh Ainy, Farid Zayeri, Rogayeh Paydar

**Affiliations:** 1 *Tobacco Control Research Center, Iranian Anti-Tobacco Association, Shahid Beheshti University of Medical Siences, Tehran, Iran. *; 2 *Department of Vice Chancellor Research Affairs, Shahid Beheshti University of Medical Sciences, Tehran, Iran.*; 3 *Proteomics Research Center and Department of Biostatistics, School of Allied Medical Sciences, Shahid Beheshti University of Medical Sciences, Tehran, Iran. *

**Keywords:** GYTS questionnaire, 7, 8 and 9 years of education, student, middle school, knowledge, behavioral intention

## Abstract

**Background and Objectives:**

Clues show that a large number of toxic agents, including carcinogenic, heavy metals, other particles, and high levels of nicotine, are effectively delivered through cigarette and hookah smoking. A pilot study was carried out in Varamin city, Iran in 2016 aimed to determine status of cigarette and hookah smoking in adolescent students using, Global Youth Tobacco Survey (GYTS) questionnaire.

**Methods:**

It was a cross-sectional study. At the first, of 63 Varamin city schools’ using cluster sampling, 48 schools were considered as primary clusters and 4 schools were selected randomly as sample clusters and students with 7, 8 and 9 years of education were studied. The total number of registered students was 1,108 and 1,075 responded to the study questionnaire. The main tool for collecting information used in this study was the GYTS questionnaire developed by the World Health Organization, was completed by subjects.

**Results:**

Totally, 479 (44.6%) students were boys and 596 (55.4%) were girls. Of 1,075 subjects the number of students at 7, 8 and 9 years education was 369 (34.3%), 362 (33.7%) and 344 (32.0%) respectively. The cigarette and hookah smoking using experience among the population was 9.2% and 25.5 respectively. Regarding the averages of the total score, although students have a relatively good attitude and specially behavioral intention (72% and 88% of the total score respectively), but only 47% of the total knowledge score by boys and 51% by girls, shows the average level of students’ awareness related the undesirable effects of smoking.

**Conclusions:**

Considering that one out of four students experienced cigarette and hookah smoking. Nearly half of the students are exposed to cigarette smoke at home or outdoors. The state of cigarette and hookah smoking in the country is alarming among school students.

## Introduction

Every 8 seconds a person in the world loses his life due to tobacco use, based on World Health Organization (WHO) study. Research results showed that starting to cigarette and hookah smoking during adolescence period (occur among more than 70 percent of cases) and if they continue for 20 years or more, they will be died 20 and 25 years earlier than people who did not have smoked in their life at all (Organization and Control, 2008; Stämpfli and Anderson, 2009). Using cigarette and hookah smoking before the age of twenty is the most critical and very dangerous dolescents; experience, it is a teenage characterizing age that affects whole person life (Amrock et al., 2015). The WHO has had extensive efforts around the world because of increasing prevalence of cigarette and hookah smoking in adolescents (Maziak et al., 2004). In the formation of personality and behavior of each individual’s a teenager’s friends play an important role during puberty period. The important point here is that when a teenager is lied in an equal age group, he tries to imitate the behavior of others, and if one or more cigarette and hookah smoking would have existed in the group, the risk of being infected is high (Lantz et al., 2013; Wills et al., 2017). On the other hand, cigarette and hookah smoking parents never can not stop their children from smoking. This is another important issue during juvenile smoking (Prokhorov et al., 2006; Abar et al., 2014). Nicotine of cigarettes is highly addictive substance and the problems could be occur if the regular access to cigarettes stopped and they feel the lack of something (O’Loughlin et al., 2002; Benowitz and Henningfield, 2013; Currie, 2016; Villanti et al., 2017). Adolescents, on the one hand, face to a number of different technologies with rapid growth and technological advancements, and on the other hand, face to a lot of mental stress in their youth period (Oksman and Turtiainen, 2004). To this reason behavioral researchers and experts believe that adolescent is more vulnerable and risky period (Steinberg, 2008; Warren et al., 2008). 

Therefore at the first, requires a complete understanding of the problem and find a solution to increase their knowledge that could be very important (Brady and Sinha, 2005; Zimmer-Gembeck and Skinner, 2011). In recent years by increasing cigarette and hookah smoking and reducing the age of onset of consumption among students, and the devastating personal, social and educational consequences, preventing of dependency is one of the serious priorities of planners and officials (Hawkins et al., 2004; Prokhorov et al., 2006). Individual members of the community must recognize and understand the existence of various problem of cigarette and hookah smoking and their risk factors, through their role in social and occupational status, will seek to cope with the issue of prevention and treatment (Tylee et al., 2007; Gerrard et al., 2008). Cigarette and hookah smoking are a reaction against the peer group and a correlation statement with the same age group (Nash et al., 2005). In the vast majority of studies on the cause of cigarette and hookah smoking, the response to the question of what caused you to use, adolescents have mentioned that unfit friends and peer groups could be as an important factor (Pedersen et al., 2007). Also, some teens have mentioned the unconscious as the cause of their drug use tendency (Brener et al., 2003). Another reason to use of cigarette and hookah smoking is rebellion against parents and a negative attitude toward their parents and adults (Brener et al., 2003). Extensive research has shown that start to cigarette and hookah smoking use in young people had meaningful relation with families who have been persistently affected by the problem, weak relation with mother and having lenient father (Hoffman et al., 2006). Children whose parents consume cigarette and hookah smoking as an introduction to addiction are more likely to be exposed to cigarette and hookah smoking (Warren et al., 2006; James et al., 2011). In order to prevent tobacco consumption, the youth situation should first be investigated for cigarette smoking, and then effective interventions should be made based on the results. The Global Youth Tobacco Survey (GYTS) questionnaire is a standard questionnaire used by most countries to determine the status of cigarette and hookah smoking in young people (Organization, 2009; Akl et al., 2010). Prevention Against Tobacco Dependence (PAD) project was used as a pilot study for the first time in the Varamin city of Iran, using the GYTS questionnaire to determine the status of cigarette and hookah smoking use among addolocents.

The results of the study will be used to planning and conduct a study at national level toward tobacco-free schools, Communities and City. 

## Materials and Methods

A pilot study was conducted using cross sectional study method in Varamin city, Iran in 2016. Number of studied population was 10,808 (5,217 female (48.3%) and 5,591 (51.7) male students. At the first, cluster sampling have been done at one-stage. Of 63 Varamin city schools’, 48 schools that were willing to cooperate with the project were considered as primary clusters (out of 48 schools, 24 were girls’ and 24 boys’ schools) and 4 schools were selected randomly as sample clusters and students with 7, 8 and 9 years of education were selected, The total number of registered students was 1,108 and 1,075 responded to the study questionnaire. Accordingly, the sample number covers about 10% of the student population. The main tool for collecting information used in this study was the GYTS questionnaire developed by the World Health Organization, which was completed by selected subjects. The questionnaire consists of a 61 core question that different communities may, depending on their cultural, religious or legal requirements, delete, change, or even add questions to it. Using questionnaire in the present study was included 65 questions, subjects field information, knowledge and attitudes about cigarette and hookah smoking, exposure to others’ hookah or cigarette smoking, knowledge of media messages about cigarette smoking and cases, teaching about cigarette and hookah smoking harm in schools, school policy questions. We added questions about students’ opinions and their perceptions of people who use cigarette and hookah smoking and the attractiveness of them for users in the viewpoint of students to the standard GYTS questionnaire. Shisha smoking, Bidi smoking and smokeless tobacco were omitted of the standard GYTS questionnaire. Questionnaire was validated using expert opinion and it has been confirmed by Cronbach’s alpha of 0.88. Knowledge, Attitude, and Behavioral Intent Questionnaire was the second tool used in this study included three sections: knowledge assessment (including 10 questions and scores range from 0 to 10), attitude (including 10 questions 4 options, the range of scores between (10 - 40) and behavioral intention (including 7 questions of 4 options with scores ranging from (7 - 28).

*Statistical analysis of data*

Since cluster sampling method was used, data were analyzed using survey data analysis. To do this, the data of the questionnaire were weighted so that the effect of sample selection (at the school level), non-response (at the student’s level), and the subsequent classification of the sample according to the distribution of gender and with 7, 8 and 9 years of education in the entire society could be considered. The weighting factor was calculated using following formula:

W=W_1_ * f_1_ * f_2_

Where in:

W_1_: Reverse the probability of choosing each school.

f_1_: Student Non-Responsiveness Modification Agent.

f_2_: Subsequent classification modifier based on gender and years number of education.

All survey analyzes were performed based on this weighting using the survey data analysis submenu in STATA software version 14.0.

## Results

In this study, a sample of 1075 students with 7, 8 and 9 years education was studied in Varamin city,Iran. Among them, 479 (44.6%) students were boys and 596 (55.4%) were girls. Table1 showed number of students at 7, 8 and 9 years education by sex. The number of students at 7, 8 and 9 years education was 369 (34.3%), 362 (33.7%) and 344 (32.0%) respectively. The age range of the subjects was 13 to 15 years. 

The cigarette and hookah smoking experience among the students was 9.2% and 25.5 respectively. Findings shows, the 9 years education was the most frequent in the students’ parents education.

Table 2 showed that what rate of students’ close friends used cigarette and hookah smoking. More than one third (36.4%) of the students acknowledge that their close friends some of the time used cigarettes or hookahs smoking. 

The proportion of girls who have tried to cigarette smoking is more than boys (9.8% vs 8.6%). Notably, the proportion of girls who had tried hookahs smoking has been more than boys (26.4% vs 24.5%). On the other hand, the proportions of boys who have ever experienced electronic cigarette smoking were two times more than girls (2.2% vs. 1.1%). Table 3 showed the age distribution of students’ first experience of cigarette or hookah smoking. The highest age prevalence of students’ first experience of cigarette or hookah smoking in boys was 12 to 13 (34.5%) and in the girls 14 to 15 (34.9%) age years old.

Table 4 describes the status of knowledge, attitude and behavioral intention of students. Regarding the averages of the total score, although students have a relatively good attitude and specially behavioral intention (72% and 88% of the total score respectively), but only 47% of the total knowledge score by boys and 51% by girls, shows the average level of students’ awareness related the undesirable effects of cigarette and hookah smoking. It is noteworthy that among students cigarettes and hookahs smoking girls have smoked more than boys all the days of the month.

## Discussion

The results showed that more than half of the students were girl and the rest were boy. The experience of cigarette and hookah smoking was 9.2% and 25.5% respectively. The level of family education in the population surveyed was the most frequent level of 9 years education among parents. More than one third of the students acknowledge that their close friends some of the time used cigarette or hookah smoking. 

The proportion of girls who have tried to cigarette and hookah smoking is more than boys. It is noteworthy that one fourth of girls have tried the hookah smoking more than boys. The first cigarette or hookah smoking experience in boys was in 12 to 13 years. It is noteworthy that among students cigarettes and hookahs smoking girls have smoked more than boys all the days of the month.

More than one third of students’ first experience of cigarette or hookah smoking in boys was 12 to 13 and in the girls 14 to 15 age years old.

The status of knowledge, attitude and behavioral intention of students shows students have a relatively good attitude and specially behavioral intention, but only 47% of the total knowledge score by boys and 51% by girls, shows the average level of students’ awareness related to the undesirable effects of cigarette and hookah smoking. In a study which was conducted by Ziaee et al., (2016) showed that there was a significant relationship between early smoking age and number of smoking days during the past 30 days. There was a meaningful relationship between the number of cigarette or hookah smoking days and the beginning of smoking under 12 years. The findings of the present study were in accordance with Ziaei et al., (2016). It was observed that 21.6% of students in Tabriz experienced a hookah smoking. For at least one-time smoking, results have shown that there was no gender difference. The study found cigarette and hookah smoking is more attractive for both sexes. Cigarette smoking is traditionally more unpleasant than hookah smoking. Age begins to drink, cigarette and hookah smoking were significant. Starting cigarette and hookah smoking was frequent among10.4 % of the students under 12 years (Khadem and Dadgarmoghaddam, 2016). 

The tobacco control program in India in 2006, based on the World Health Organization (WHO), has shown that tobacco use among girls in some parts of India was increased and alarming. Tobacco control is one of the subjects that should not be overlooked, and this issue needs special attention among school staffs. A high prevalence of tobacco use among school staff, students and especially among girl’s students in Iran is alarming. Reducing the rate of cigarette smoking and exposure to secondhand smoke among students should be more emphasized (SInha, 2008). 

Indonesia’s Global Tobacco and Youth Report (GYTS) in 2014 also showed that 20.3% of schoolchildren used cigarette smoking (36.2% boys, 4.4% girls). Active smokers were 3.18%, of who smoke every day 35.6%. Among 43.2% of students cigarette smoking started at the age of 12 to 13 years (Organization, 2015). The World Tobacco Study in Gambia in 2008 showed that there were no statistically significant differences in the gender pattern of tobacco consumption in the Gambia. It is noteworthy that a large percentage of students, especially girls, used other tobacco products. This issue needs to be further addressed (Martinasek et al., 2011; Organization, 2013). 

In the present study, hookah smoking consumption was high among students and even hookah smoking consumption in high school girls was higher than boys. The findings of this study also were in line with findings of the study in Indonesia and Gambia. Considering that one out of four students experienced cigarette and hookah smoking, the state of cigarette and hookah smoking in the country especially among girls (13-14 years) is alarming. On the other hand, the pattern of cigarette and hookah smoking in our country’s teens was similar to that of other countries.

**Table 1:** Distribution of Students at 7, 8 and 9 Year’s Education by Sex

Education (years)	Boys	Girls	Total
7	130 (12.1)	239 (22.2)	369 (34.3)
8	187 (17.4)	175 (16.3)	362 (33.7)
9	162 (15.1)	182 (16.9)	344 (32.0)
Total	479 (44.6)	596 (55.4)	1,075 (100)

**Table 2:** Distribution of Cigarette or Hookah Smoking Using by Students’ Close Friends

Cigarette or hookah Smoking using by students’ close friends	Number (percent)
None	599 (55.7)
Some	391 (36.4)
Most	59 (5.5)
All	26 (2.4)

**Table 3:** Distribution and Confidence Interval of Students’ Age at First Experience of Cigarette or Hookah Smoking

Answer (year)	Boys Percent (confidence interval)	Girls Percent (confidence interval)	Total Percent (confidence interval)
<7	11.8 (7.9-17.3)	7.4 (4.9-10.9)	71.9 (62.1-80.1)
9-Aug	8.7 (2.8-23.6)	9.9 (3.3-26.4)	9.4 (5.3-16.1)
11-Oct	15.7 (7.8-29.1)	24.6 (21.0-28.6)	9.4 (4.1-19.9)
13-Dec	34.5 (26.2-44.0)	23.2 (21.8-24.6)	20.5 (12.0-32.7)
14-15	29.3 (23.5-35.8)	34.9 (25.7-45.4)	28.5 (18.4-41.3)

**Table 4:** Descriptive Statistics Status of Students' Knowledge, Attitude and Behavioral Intention

Gender	Variable	Range of Changes	Average ratio of total score	Mean (SD)	Confidence interval 95%
Boys	knowledge	0-8	0.47	3.72±0.10	(3.40-4.03)
	attitude	16-40	0.74	29.73±0.20	(29.09-30.36)
	behavioral intention	7-28	0.9	25.18±0.29	(24.28-26.09)
Girls	knowledge	0-8	0.51	4.10±0.13	(3.68-4.51)
	attitude	12-40	0.71	28.21±0.14	(27.77-28.65)
	behavioral intention	7-28	0.86	24.16±0.48	(22.64-25.68)
Total	knowledge	0-8	0.49	3.93±0.14	(3.50-4.36)
	attitude	12-40	0.72	28.88±0.43	(27.51-30.25)
	behavioral intention	7-28	0.88	24.69±0.41	(23.30-25.92)

## Team Work

Ayatollah Seyyed Morteza Mahmoudi Golpayegani, Imam Jumah, Governor, Head of Education, Managers and Teachers of Ammar High Schools, Velayat, Azadgan and Tajik City of Varamin. Habib Emami, Hooman Sharifi, Ali Abdollahi Nia, Nasir Dehghan, Ali Ata Taheri, Hamid Reza Shahsavan, Rahmatollah Shahramifar, Nader Yazdanpanahi, Hossein Alipour, Ahmad Hamed, Siavash Khodaei, Fatemeh Matinkhah, Sonia Ghaffari, Elaham Maoff, Zahra Sadr, Mana Zahedi, Leyla Morsali, Zainab Vaziri, Nahid Sarabi, Azadeh Nazari, Marjan Nemati, Masoumeh Gordati, Atefeh Khaleqi, Nayereh Elahi, Fatemeh Bahrami, Sanaz Hamzeali.
